# Morphological Remodeling of Scalp High-Frequency Oscillations Across BASED-Stratified Groups in Infantile Epileptic Spasms Syndrome

**DOI:** 10.3390/diagnostics16132024

**Published:** 2026-06-29

**Authors:** Keisuke Maeda, Shunta Yamaguchi, Himari Tsuboi, Naohiro Ichino, Keisuke Osakabe, Keiko Sugimoto, Gen Furukawa, Naoko Ishihara

**Affiliations:** 1 Department of Clinical Physiology, Fujita Health University School of Medical Sciences, 1-98 Dengakugakubo, Kutsukake-cho, Toyoake 470-1192, Japan; 2 Department of Clinical Laboratory, Fujita Health University Hospital, 1-98 Dengakugakubo, Kutsukake-cho, Toyoake 470-1192, Japan; 3 Department of Medical Sciences Education, Fujita Health University School of Medical Sciences, 1-98 Dengakugakubo, Kutsukake-cho, Toyoake 470-1192, Japan; 4 Department of Pediatrics, Fujita Health University School of Medicine, 1-98 Dengakugakubo, Kutsukake-cho, Toyoake 470-1192, Japan

**Keywords:** high-frequency oscillations (HFOs), epilepsy, infantile epileptic spasms syndrome (IESS), biomarker, electroencephalography (EEG), Burden of Amplitudes and Epileptiform Discharges (BASED) score, morphological analysis, phenotypic stratification

## Abstract

**Background/Objectives**: High-frequency oscillations (HFOs)—transient electroencephalography (EEG) activity above 80 Hz—are emerging biomarkers of infantile epileptic spasms syndrome (IESS). However, the relationship between their multidimensional characteristics and clinical severity remains poorly understood. This study aimed to clarify the association of scalp HFO morphology with severity across levels defined by the Burden of Amplitudes and Epileptiform Discharges (BASED) score, an interictal EEG grading scale for IESS. **Methods**: We enrolled 53 children with epilepsy (30 with IESS and 23 non-IESS controls) and quantified HFO frequency, duration, amplitude, and cycle count from automatically detected scalp HFOs during interictal EEG. **Results**: Patient-level median analyses demonstrated significant monotonic associations with BASED severity: HFO frequency decreased (Spearman *ρ* = −0.46, *p* = 0.001) and duration increased (*ρ* = 0.32, *p* = 0.026). Event-level mixed-effects models confirmed these findings, showing that frequency decreased by 10.6 Hz per BASED step (*p* < 0.001) and duration increased 1.18-fold per step (*p* = 0.011), whereas amplitude and cycle count showed no consistent associations. Phenotype-level enrichment analysis revealed that specific morphological signatures significantly distinguished severity levels, with severe IESS showing a marked reduction in the high-frequency/high-amplitude/short-duration class (OR = 0.49, 95% CI 0.33–0.73) and a shift toward low-frequency/long-duration phenotypes. **Conclusions**: Scalp HFOs showed lower frequencies and longer durations in higher BASED-stratified groups, suggesting that HFO morphology may provide quantitative information complementary to visual EEG assessment in IESS. These findings support the potential utility of HFO phenotypic stratification for objective evaluation and longitudinal monitoring of disease burden.

## 1. Introduction

Infantile epileptic spasms syndrome (IESS) is a severe developmental and epileptic encephalopathy of infantile onset, characterized by epileptic spasms, a distinctive interictal electroencephalography (EEG) pattern, and developmental plateauing or regression [1,2]. The syndrome encompasses the classic West syndrome triad as well as incomplete presentations, and affects approximately 2–4 per 10,000 live births, typically manifesting between 3 and 12 months of age [1]. The severity of interictal epileptiform activity on EEG is recognized as a key determinant of both treatment response and neurodevelopmental prognosis, underscoring the clinical importance of reliable and objective EEG-based severity assessment.

Hypsarrhythmia, the classically described interictal EEG pattern of high-amplitude, chaotic slow waves intermixed with multifocal spikes, has long served as the electrographic hallmark of IESS. However, it is subject to poor inter-rater reliability and may be absent in a substantial proportion of patients [3]. To address these limitations, Mytinger and colleagues proposed the Burden of Amplitudes and Epileptiform Discharges (BASED) score, an interictal EEG grading scale revised in 2021 that rates EEG severity from 0 (normal) to 5 (definite epileptic encephalopathy) and demonstrates moderate-to-high inter-rater reliability [4,5]. However, the BASED score remains fundamentally dependent on visual EEG interpretation, and residual inter-rater variability persists even with formal rater training [6], highlighting the need for automated or semi-automated quantitative EEG biomarkers that can complement visual severity grading.

High-frequency oscillations (HFOs) are transient EEG events with frequencies exceeding 80 Hz, encompassing the ripple band (80–250 Hz), and have been established as biomarkers of epileptogenicity through intracranial EEG research [7,8,9,10,11,12,13,14,15,16,17,18,19,20,21,22]. Importantly, HFOs are also detectable non-invasively via scalp EEG and have been shown to outperform spikes in localizing the epileptogenic zone, and to decrease following successful treatment [7]. In IESS specifically, interictal scalp HFO rates are significantly higher in patients with active epileptic spasms than in those without [12,23], and exhibit a dose–response relationship with BASED-defined EEG severity [24]. These findings indicate that scalp HFOs capture severity-related changes in epileptic activity.

However, prior investigations in IESS have focused predominantly on HFO detection rates, with little attention paid to the morphological characteristics of individual HFO events, including their dominant frequency, duration, amplitude, and cycle count. In other epilepsy contexts, HFO morphology has proven clinically informative: epileptogenic HFOs recorded intracranially exhibit distinct amplitude profiles compared to non-epileptogenic oscillations [25], and scalp HFO phenotypic clustering based on multidimensional morphological features has identified subclasses that differentially predict clinical disease states [26]. These observations suggest that HFO morphology may encode underlying pathophysiological changes, but whether scalp HFO characteristics differ systematically across BASED-stratified groups in IESS remains unclear.

The present study aimed to characterize severity-associated alterations in scalp HFO morphology across BASED-defined EEG severity levels in children with IESS and non-IESS controls. We applied patient-, event-, and phenotype-level analytical frameworks to examine how HFO characteristics relate to interictal EEG severity. We specifically sought to determine whether multidimensional scalp HFO morphology could serve as an objective marker of disease severity that complements visual EEG assessment in IESS.

## 2. Materials and Methods

### 2.1. Study Participants

This retrospective study included 53 children with epilepsy who underwent routine scalp EEG recordings at Fujita Health University Hospital between 2018 and 2024. Of these, 30 patients (20 males, 10 females) had a clinical diagnosis of IESS by a child neurologist, and 23 patients (10 males, 13 females) served as age-matched non-IESS controls. IESS was diagnosed according to the 2022 International League Against Epilepsy (ILAE) criteria [1,27] by specialists in clinical epilepsy who were blinded to the HFO analysis. The non-IESS control group was clinically heterogeneous and included children with focal or generalized epilepsy, those evaluated for suspected epilepsy without a definitive diagnosis, and patients in a post-IESS state. For each patient, one scalp EEG recording was selected if it met the following inclusion criteria: (1) sampling frequency ≥ 1000 Hz; (2) recorded during interictal (non-seizure) periods; and (3) containing sufficient non-rapid eye movement (NREM) sleep to support stable HFO detection. In this study, “interictal” was operationally defined as an EEG segment containing no clinical spasms or electrographic seizures. Clinical data including sex, age at EEG recording, age at IESS onset, and treatment status were collected through a detailed chart review. EEG timing was determined by clinical indication rather than a uniform treatment window.

This study adhered to the principles of the Declaration of Helsinki [28] and its later amendments and was approved by the Ethics Committee of Fujita Health University (Approval No. HM22-143). Patient consent was obtained using an opt-out method posted on the university website.

### 2.2. Scalp EEG Recording and Preprocessing

Scalp EEG recordings were obtained using the Neurofax system (Nihon Kohden, Tokyo, Japan) with 19 electrodes placed according to the international 10–20 system (Fp1, Fp2, F3, F4, C3, C4, P3, P4, O1, O2, F7, F8, T3, T4, T5, T6, Fz, Cz, Pz) and sampled at 1000 Hz to ensure adequate bandwidth for HFO analysis. Signals were re-referenced to a common-average montage, and subsequent analyses were performed using these 19 derivations. This re-referencing was applied only for automated HFO detection and morphometric analysis. In contrast, the BASED score was applied within its original clinical framework using a longitudinal bipolar montage as an external index of EEG burden. Preprocessing involved visual inspection for gross artifacts and exclusion of segments contaminated by movement, muscle activity, or electrode disturbances. An artifact-free, approximately 600 s segment of NREM sleep EEG was selected by a specialist technician certified by the Japanese Society of Clinical Neurophysiology, who was blinded to all clinical information. NREM sleep was identified by visual inspection by a specialist technician certified by the Japanese Society of Clinical Neurophysiology, who was blinded to all clinical information.

### 2.3. Automatic Detection of Scalp HFOs

**Figure 1:**
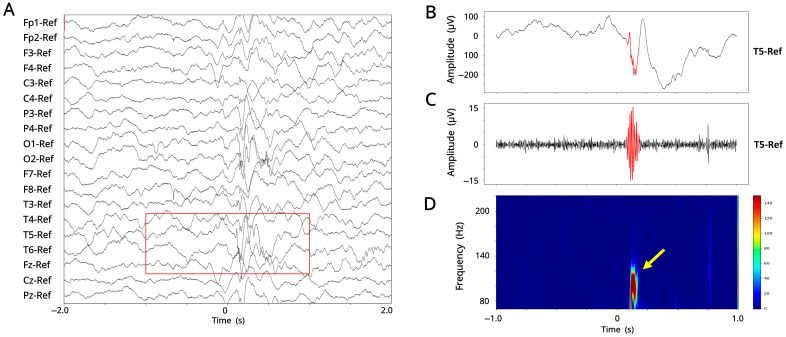
Representative example of scalp HFO detection. (**A**) Raw scalp EEG (0.5–300 Hz) demonstrating a transient high-frequency burst within the multichannel recording. The red box denotes the segment selected for further analysis. (**B**) Zoomed raw EEG segment from T5-Ref. The red trace indicates the candidate HFO event. (**C**) Ripple-band filtered signal (80–250 Hz), with the red trace highlighting the corresponding ripple oscillation. (**D**) Time–frequency spectrogram of the same event, showing a focal increase in spectral power within the ripple band (80–200 Hz; yellow arrow). Time–frequency representations were computed using Morlet wavelets. The color scale indicates the relative power change (%) compared to the baseline period (1.0–1.2 s). This example illustrates the typical morphology of scalp HFOs analyzed in this study and the multistep processing workflow used for their detection and characterization.

Scalp HFOs were defined as oscillatory events consisting of at least four sinusoidal-like oscillations with a dominant frequency between 80 and 250 Hz that clearly stood out from the background EEG activity. HFOs were automatically detected within the 80–250 Hz band using the Hilbert Detector proposed by Crépon et al. [29] and subsequently underwent blinded visual validation by a certified specialist technician. The detector operates in two stages: first, the EEG signal is bandpass-filtered to the frequency range of interest and the analytic amplitude (envelope) is extracted using the Hilbert transform; second, candidate HFO events are identified by detecting local maxima of the envelope exceeding a threshold set to five times the standard deviation of the envelope computed across the entire EEG recording. Automatically detected HFOs were then visually reviewed, and events contaminated by non-physiological noise or muscle activity were excluded [30]. This Hilbert-based approach was used as a single, consistent detection framework across all recordings to enable standardized extraction of scalp HFO characteristics for quantitative analysis. It was selected for the present study because our primary objective was not to compare detectors, but to examine the association between BASED-defined EEG burden and uniformly quantified scalp HFO characteristics within the same dataset. Figure 1 illustrates the morphology of automatically detected scalp HFOs and the processing steps performed in a representative example from channel T5.

### 2.4. Extraction of HFO Characteristics

For each detected HFO event, four characteristics were quantified:

• Frequency (Hz): the average oscillation frequency computed from Hilbert-transform-based peak spacing;
• Duration (ms): the time interval between the onset and offset of the event;
• Amplitude (Z-score): the peak-to-peak intensity normalized within each EEG channel;
• Cycle count (cycles): the total number of oscillatory cycles contained within the event.

For patient-level analyses, median values per subject were used to represent each characteristic. For event-level analyses, all HFOs were treated as individual observations with patient-clustered statistical models. For phenotype-level analyses, frequency, amplitude, and duration were binarized at their global medians to generate eight phenotypic classes representing all combinations of high/low frequency, high/low amplitude, and short/long duration. This median-based dichotomization was adopted to provide a clinically interpretable framework for exploratory analysis while avoiding excessive subdivision of event classes.

### 2.5. BASED Scoring

The BASED score is an interictal EEG grading scale originally proposed in 2015 [4] and revised in 2021 by Mytinger et al. [5]. In the present study, all interictal EEG recordings were graded using the revised 2021 criteria. The scoring criteria are shown in the Supplementary Materials (Supplementary Table S1). Scores were assigned in randomized order by blinded reviewers consisting of specialist technicians certified by the Japanese Society of Clinical Neurophysiology.

The BASED scale ranges from 0 (normal) to 5 (most severe). Scoring was based on the most epileptiform 5 min epoch of NREM sleep; if criteria for scores 3–5 were not met within this epoch, scores of 0–2 were applied based on the overall EEG background. For subsequent analyses, BASED scores were categorized into three severity groups (≤3, 4, and 5) representing increasing interictal epileptic burden. Although HFO morphology and BASED scores were derived from the same EEG recordings, they represent distinct analytical measures: automated, event-based quantitative descriptors versus visual EEG severity grading.

### 2.6. Statistical Analysis

Variables with log-normal distributions were summarized as medians with interquartile ranges (IQRs), whereas variables approximating normal distributions were expressed as means ± standard deviations (SDs).

Three complementary statistical approaches were employed to evaluate associations between HFO characteristics and BASED-defined EEG severity. First, patient-level analysis assessed monotonic associations using Spearman rank correlation with 10,000 permutation-based *p*-values, with pairwise comparisons between the ≤3, 4, and 5 severity groups performed using the Brunner–Munzel test, supplemented with Hodges–Lehmann median differences and Cliff’s δ effect sizes. Multiple comparisons were adjusted using the Benjamini–Hochberg false discovery rate (FDR) procedure. Second, event-level differences in HFO characteristics across BASED scores were quantified using linear mixed-effects models with patient ID as a random intercept and age as a covariate. Duration and cycle count were log-transformed to satisfy model assumptions, and results were expressed as per-step differences (frequency) or per-step multipliers (duration and cycle count). Third, phenotype-level analysis evaluated enrichment of each of the eight HFO phenotypic classes using generalized estimating equations with a binomial link and patient-clustered exchangeable covariance structure, reporting odds ratios per BASED step with 95% confidence intervals.

All analyses were performed using Python version 3.13 (statsmodels, scipy, numpy) and JMP version 18 (SAS Institute, Inc., Cary, NC, USA), with statistical significance defined as *p* < 0.05 or FDR-adjusted thresholds, as appropriate.

## 3. Results

### 3.1. Basic Characteristics

A total of 53 children with epilepsy were included: 30 with IESS and 23 non-IESS controls. Among the IESS group, 20 were male and 10 were female; the non-IESS control group comprised 10 males and 13 females. The mean age at EEG recording was 2.3 (1.3–6.2) years in the IESS group and 2.0 (0.8–5.6) years in the non-IESS control group. According to the BASED severity classification, 23 patients were categorized as BASED ≤3, 9 as BASED 4, and 21 as BASED 5. After artifact rejection and preprocessing, 684 scalp HFO events were detected in the IESS group and 144 events in the non-IESS control group (Table 1).

### 3.2. Patient-Level Associations Between HFO Characteristics and BASED Severity

**Figure 2:**
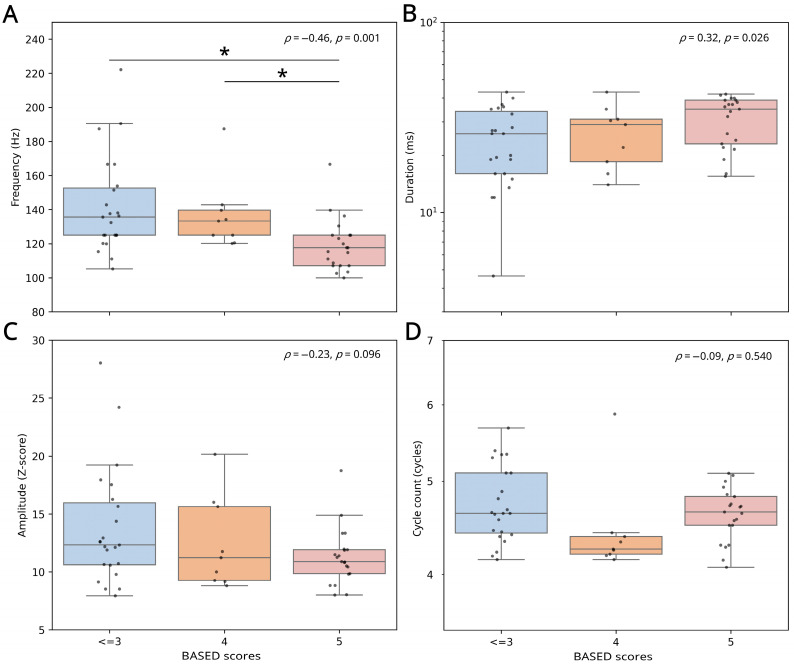
Patient-level scalp HFO characteristics across BASED severity groups. Box plots show the distribution of median HFO frequency (**A**), duration (**B**), amplitude (**C**), and cycle count (**D**) for each patient, stratified by BASED severity group (≤3, 4, and 5). Each dot represents an individual patient’s median value. Spearman rank correlation coefficients (*ρ*) and permutation-based *p*-values are shown in each panel. * *p* < 0.01 (Brunner–Munzel tests with Benjamini–Hochberg false discovery rate correction). Abbreviations: BASED score, burden of amplitudes and epileptiform discharges score.

Patient-level median HFO characteristics demonstrated significant monotonic associations with BASED severity (Figure 2). Frequency showed a significant negative association (Spearman *ρ* = −0.46, *p* = 0.001), while duration showed a significant positive association (*ρ* = 0.32, *p* = 0.026). Amplitude (*ρ* = −0.23, *p* = 0.096) and cycle count (*ρ* = −0.09, *p* = 0.540) did not exhibit statistically significant monotonic trends.

Pairwise Brunner–Munzel comparisons further clarified these severity-related differences. For frequency, the BASED ≤ 3 vs. 5 comparison yielded a Hodges–Lehmann (HL) difference of 17.86 Hz (FDR-adjusted *p* = 0.002), and the BASED 4 vs. 5 comparison yielded an HL difference of 15.68 Hz (FDR-adjusted *p* = 0.002). For duration (log-scale), the BASED ≤ 3 vs. 5 comparison showed a weak HL difference of −0.27, but this did not reach statistical significance after FDR adjustment (*p* = 0.08). Amplitude and cycle count showed no consistent pairwise differences.

In the within-IESS analysis comparing BASED 4 and 5, frequency remained lower in the more severe group, whereas duration showed only a non-significant trend toward prolongation (Supplementary Table S2).

### 3.3. Event-Level Modeling of BASED-Related Changes in HFO Characteristics

Event-level mixed-effects modeling confirmed BASED-related differences in HFO morphology (Table 2). Frequency decreased significantly with increasing severity (*β* = −10.56 Hz per BASED step, 95% CI −16.62 to −4.50, *p* < 0.001). Duration increased with severity, reflected by a per-step multiplier of 1.178 (95% CI 1.039–1.335, *p* = 0.011). Amplitude showed no significant association (*β* = −0.49, 95% CI −1.32 to 0.35, *p* = 0.252), and cycle count likewise exhibited no severity-related change (per-step multiplier = 1.001, 95% CI 0.981–1.020, *p* = 0.941). Importantly, the direction of the event-level findings was concordant with that of the patient-level analysis, in which each subject contributed a single summary measure rather than all detected events; in both analytical frameworks, higher BASED scores were associated with lower scalp HFO frequency and longer duration.

We then performed an additional within-IESS event-level analysis restricted to BASED 4 and 5 cases. Consistent with the full-cohort model, scalp HFO frequency remained lower in the BASED 5 group, whereas duration showed a concordant but non-significant trend toward prolongation (Supplementary Table S3).

### 3.4. Phenotypic Enrichment Analysis of HFO Morphologies Across Severity Levels

**Figure 3:**
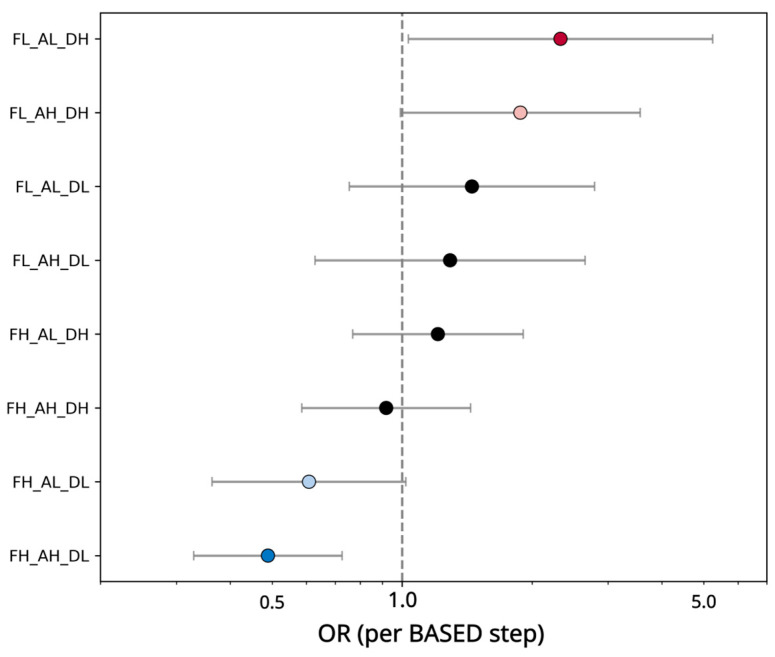
Phenotype-level enrichment of scalp HFO morphological classes across BASED severity levels. Forest plot showing ORs per BASED step with 95% CIs for each of the eight binarized HFO phenotypic classes, derived from generalized estimating equation models. Each class represents a combination of high (H) or low (L) frequency (F), amplitude (A), and duration (D), binarized at their respective global medians. Classes are ordered by OR. The vertical dashed line indicates OR = 1 (no association). Dark red and dark blue symbols denote statistically significant increases or decreases, respectively (FDR-adjusted *p* < 0.05); light red and light blue symbols indicate borderline associations; black symbols denote non-significant findings. Abbreviations: BASED, burden of amplitudes and epileptiform discharges; OR, odds ratio.

Phenotype-level analysis using the eight phenotypic classes revealed significant severity-related alterations in HFO morphology (Figure 3). The high-frequency × high-amplitude × short-duration (FH_AH_DL) class showed a strong negative association with the BASED score (odds ratio [OR] = 0.49, 95% CI 0.33–0.73, *p* < 0.001). Conversely, several long-duration phenotypes demonstrated increased probabilities with increasing severity. The low-frequency × low-amplitude × long-duration (FL_AL_DH) class was significantly enriched (OR = 2.33, 95% CI 1.03–5.24, *p* = 0.041), whereas the low-frequency × high-amplitude × long-duration (FL_AH_DH) class showed a borderline trend (OR = 1.88, 95% CI 0.99–3.56, *p* = 0.053). A similar borderline pattern was observed for the high-frequency × low-amplitude × short-duration (FH_AL_DL) class (OR = 0.61, 95% CI 0.36–1.02, *p* = 0.060). No statistically significant associations were observed for the remaining phenotypes (FH_AH_DH, FL_AH_DL, and FL_AL_DL).

## 4. Discussion

This study demonstrated that scalp HFO morphology differed systematically across BASED-stratified groups in this cohort. Specifically, higher BASED scores were associated with lower HFO frequencies and longer durations, whereas amplitude and cycle count showed no consistent trends. At the phenotypic level, higher-score groups showed relative depletion of the high-frequency × high-amplitude × short-duration class and enrichment of low-frequency/long-duration phenotypes, suggesting a shift toward a “slower and more prolonged” HFO morphological profile. These findings show that severity-related morphological patterns can be captured noninvasively on scalp EEG using multidimensional HFO phenotyping rather than HFO detection rate alone. However, because the analyzed scalp HFOs may have included both physiological and pathological events, the results should be interpreted as shifts in the overall distribution of detected HFO events across EEG severity strata rather than as definitive evidence of intrinsic morphological transformation of individual pathological HFOs.

The reduction in HFO frequency with increasing severity is consistent with intracranial observations showing that interictal pathological HFOs have lower mean frequencies than physiological oscillations [31]. This pattern suggests that as severity increases, an increasing proportion of scalp-detectable HFOs arises from pathological generators rather than normal cortical activity. One plausible mechanism is that progressive excitation–inhibition imbalance and cortical hyperexcitability underlying higher BASED scores disrupt the precise neuronal synchrony required to sustain high-frequency ripple activity, resulting in a downward shift in dominant oscillatory frequency [32,33].

The severity-related increase in HFO duration similarly aligns with the pathological HFO phenotype established in intracranial studies, which have shown that pathological events exhibit significantly longer durations than their physiological counterparts [31]. Prolonged duration may reflect impaired oscillatory termination within a hyperexcitable IESS cortex [32], consistent with the 1.18-fold per-step increase observed independently at both patient and event levels, thereby enabling epileptiform bursts to persist beyond the brief events that typify physiological ripples.

HFO amplitude and cycle count did not show consistent associations with BASED severity. For amplitude, two factors are relevant: within-channel Z-score normalization removes between-patient variation in absolute signal levels (which would be necessary to detect systematic severity differences), and scalp-recordings are further attenuated by the skull and soft tissues, with individual anatomical variation introducing noise that may obscure true severity-related differences [7]. For cycle count, the absence of an effect is expected because it approximates the product of duration and frequency, which change in opposite directions, effectively canceling each other and yielding the near-unity per-step multiplier observed in the model. These null findings suggest that frequency and duration may be more informative markers of IESS severity than amplitude or cycle count.

The phenotype-level enrichment analysis provided a clinically interpretable complement to the dimensional findings. The high-frequency × high-amplitude × short-duration phenotype (FH_AH_DL), markedly depleted in severe IESS (OR = 0.49), closely resembles physiological-type HFOs described in the normal human brain; i.e., brief, high-frequency oscillations associated with routine cortical processing [31,34]. Its progressive reduction suggests that normal cortical oscillatory activity may be increasingly displaced by pathological-type events across higher BASED-defined EEG severity strata in this cohort. Conversely, low-frequency × long-duration phenotypes became enriched, corroborating the individual-dimension findings. A comparable phenotypic stratification approach applied to scalp HFOs in absence epilepsy similarly identified low-frequency and long-duration clusters as differential predictors of ictal activity [26], suggesting the broader relevance of multidimensional HFO characterization across epilepsy syndromes. These phenotypic shifts may reflect a relative enrichment of scalp HFO patterns more commonly associated with pathological activity across higher BASED-defined EEG burden strata, rather than a definitive conversion of physiological HFOs into pathological HFOs.

From a clinical perspective, these findings suggest that scalp HFO morphology could serve as a quantitative biomarker of the underlying epileptic network in infants. Unlike traditional visual EEG grading, which is prone to inter-rater variability, morphology-based metrics derived from automated detection could offer a reproducible approach to assessing disease burden. Because the 2021 BASED framework was developed using longitudinal bipolar montage, whereas scalp HFO characteristics were quantified here using a common-average reference, direct comparability is limited. Accordingly, the present HFO metrics should be interpreted as complementary quantitative markers associated with BASED-defined EEG burden rather than as direct equivalents of the amplitude-based BASED criteria. Future integration of these morphological signatures into diagnostic pipelines may enable real-time quantitative monitoring of treatment response, potentially facilitating earlier clinical interventions.

This study has several limitations. First, the retrospective, single-center design and moderate sample size, particularly the small BASED 4 subgroup (*n* = 9), limit statistical power and generalizability; external validation in larger multi-center cohorts is required. In addition, because lower BASED scores were sparsely represented among IESS cases without hypsarrhythmia, non-IESS cases were included to preserve coverage of the lower score range. This introduces clinical heterogeneity into the lower BASED strata and may have affected group-level comparisons; accordingly, the full-cohort findings should be interpreted with caution. Second, the cross-sectional design precludes causal inference about the temporal relationship between morphological remodeling and clinical severity. Third, within-channel Z-score amplitude normalization may have reduced sensitivity to between-patient severity-related differences, and alternative normalization strategies merit future investigation. Fourth, although age was included as a covariate in event-level models, residual developmental confounding on HFO characteristics cannot be entirely excluded. Fifth, treatment status and the use of sleep-inducing agents may also have influenced HFO morphology, and these effects could not be fully separated from disease-related effects in this retrospective cohort. Sixth, because HFO morphology and BASED scores were derived from the same EEG recordings, the findings should be interpreted as within-recording associations rather than as relationships between fully independent measures. Seventh, the overlap between diagnostic groups and severity strata means that the full-cohort analysis should be interpreted primarily as a comparison across BASED-stratified groups, whereas the within-IESS analysis provides more limited support for a severity-based interpretation. Eighth, the Hilbert-based detector likely captured both physiological and pathological HFOs, and the current dataset did not allow reliable separation of these event types. Ninth, the use of a common-average reference for HFO analysis, in contrast to the longitudinal bipolar montage used in the original BASED framework, limits direct comparability between these measures. Finally, because cross-detector validation was not performed and EEG timing was not standardized with respect to treatment phase or disease progression, detector-dependent and longitudinal effects cannot be excluded. Future prospective longitudinal studies incorporating spike-coupling and spatial-context analyses will be important to validate and extend these findings.

## 5. Conclusions

In conclusion, scalp HFOs in IESS showed systematic morphological differences across BASED-stratified groups. The most prominent findings were a shift toward lower frequencies and longer durations in groups with higher BASED scores. Although the biological interpretation requires caution because the present study did not distinguish pathological from physiological HFOs, these results suggest that HFO morphology may provide a valuable quantitative complement to visual EEG assessment. Incorporation of morphology-based metrics into analytical frameworks may support objective assessment and longitudinal monitoring of disease burden in IESS.

## Figures and Tables

**Table 1:** Clinical and electroencephalographic characteristics.

	Non-IESS (*n* = 23 )	IESS (*n* = 30)	*p*
Sex (*n*, %)			
Male	10 (43.5)	20 (66.7)	0.09 ^‡^
Female	13 (56.5)	10 (33.3)	
Age at the time of EEG recording (years) *	2.0 (0.8–5.6)	2.3 (1.3–6.2)	0.54 ^§^
Age at IESS onset (years) *	–	0.0 (0.0–0.0)	–
IESS duration (day) ^†^	–	1970.0 ± 1533.7	–
Medication (*n*, %)	13 (56.5)	26 (86.7)	0.01 ^‡^
Clinical category of non-IESS (*n*, %)			
Suspected epilepsy/no confirmed epilepsy	3 (13.0)	–	–
Focal epilepsy	7 (30.5)	–	
Generalized epilepsy	4 (17.4)	–	
Post-IESS	9 (39.1)	–	
Sleep-inducing agent user during EEG recording (*n*, %)	11 (47.8)	6 (20.0)	0.03 ^‡^
Analyzed EEG recording duration (sec) *	571.0 (500.0–600.0)	601.0 (569.5–601.0)	0.44 ^§^
BASED score (*n*, %)			
0	3 (13.0)	0 (0.0)	<0.001 ^‡^
1	8 (34.8)	0 (0.0)	
2	3 (13.0)	0 (0.0)	
3	9 (39.1)	0 (0.0)	
4	0 (0.0)	9 (30.0)	
5	0 (0.0)	21 (70.0)	
Scalp HFO events (*n*)	144	684	
Scalp HFO detection rates (detections/min) *	0.4 (0.1–0.9)	1.2 (0.6–2.8)	0.003 ^§^

Abbreviations: BASED score, burden of amplitudes and epileptiform discharges score; EEG, electroencephalography; HFO, high-frequency oscillation; IESS, infantile epileptic spasms syndrome. * Data are expressed as median (25th–75th percentiles). ^†^ Data are expressed as mean ± SD. ^‡^ χ^2^ test. ^§^ Wilcoxon rank-sum test.

**Table 2:** Event-level mixed-effects model estimates for the association between BASED score and scalp HFO characteristics.

Scalp HFO Characteristic	Estimate (per BASED Step) *	95% CI	*p * ^§^	FDR-Adjusted *p *^‖^
Frequency (Hz)	−10.56 ^†^	−16.62 to −4.50	<0.001	0.003
Duration (fold-change)	1.178 ^‡^	1.039–1.335	0.011	0.021
Amplitude (Z-score)	−0.49 ^†^	−1.32 to 0.35	0.252	0.336
Cycle count (fold-change)	1.001 ^‡^	0.981–1.020	0.941	0.941

Abbreviations: BASED, burden of amplitudes and epileptiform discharges; CI, confidence interval; FDR, false discovery rate; HFO, high-frequency oscillations. * Estimates represent the effect of a one-step increase in BASED score (ordinal). ^†^ Estimates are expressed as absolute differences (Hz, Z-score). ^‡^ The mixed-effects model fitted on a log-transformed scale; therefore, estimates are expressed as multiplicative ratios after exponentiation (e.g., 1.178 corresponds to a 17.8% increase per BASED step). ^§^ *p*-values are derived from linear mixed-effects models with patient-level random intercepts and age as a covariate. ^‖^ FDR-adjusted *p*-values reflect Benjamini–Hochberg correction applied within this table to account for multiple comparisons across the four HFO characteristics.

## Data Availability

The data presented in this study are available on request from the corresponding author due to ethical restrictions.

## Supplementary Materials

The following supporting information can be downloaded at: https://www.mdpi.com/article/10.3390/diagnostics16132024/s1, Table S1: The 2021 BASED score. Table S2: Patient-level comparison of scalp HFO characteristics between BASED 4 and 5 within the IESS cohort. Table S3: Event-level comparison of scalp HFO characteristics between BASED 4 and 5 within the IESS cohort.

## Abbreviations

The following abbreviations are used in this manuscript:

BASED	Burden of AmplitudeS and Epileptiform Discharges
CI	Confidence interval
EEG	Electroencephalography
FDR	False discovery rate
GEE	Generalized estimating equations
HFO	High-frequency oscillations
HL	Hodges–Lehmann
IESS	Infantile epileptic spasms syndrome
ILAE	International League Against Epilepsy
IQR	Interquartile range
NREM	Non-rapid eye movement
OR	Odds ratio
SD	Standard deviation
