# Supplementary material for: Morphological Remodeling of Scalp High-Frequency Oscillations Across BASED-Stratified Groups in Infantile Epileptic Spasms Syndrome
Source: Diagnostics (Basel). 2026 Jun 29;16(13):2024. doi: 10.3390/diagnostics16132024 (PMC13360615; doi:10.3390/diagnostics16132024)
Supplement: Supplementary file 1 [file diagnostics-16-02024-s001.zip › diagnostics-4258442-supplementary.pdf]

**Supplementary Table S1.** The 2021 BASED score

| BASED score | Description |
|---|---|
| **0** | Normal |
| **1** | Any definite non-epileptiform abnormality |
| **2** | <3 spike foci **AND** no channel with abnormal high amplitude |
| **3** | >3 spike foci <50% of one-second bins **AND** no channel with abnormal high amplitude, **OR** |
| | <3 spike foci but >1 channel with abnormal high amplitude |
| **4 (Probable EE)** | ≥3 spike foci <50% of one-second bins **AND** ≥1 channel with abnormal high amplitude, **OR** |
| | Not meeting criteria for 5 but includes GMFS or paroxysmal voltage attenuations |
| **5 (Definite EE)** | ≥3 spike foci that are ≥50% of one second bins |

Abbreviations: BASED: Burden of AmplitudeS and Epileptiform Discharges, GMFS: grouped multifocal spikes, EE: epileptic encephalopathy.

**BASED Score Rules:** Apply score 3–5 to the most epileptic 5 min epoch; if no score is reached, apply score 0–2 to the remainder of the study

**>3 Spike Foci Rules:**

1. Maybe at least one from each hemisphere OR all from one hemisphere (may include midline)
2. If >3 spike foci in the entire study but no 3 spike foci within 5 min, and no channel with abnormally high amplitude, the BASED score is 2 + uncommon multifocal spikes

**Spike Burden Rules:**

1. % one-second bins that include 1 or more spikes in the most epileptic 5 min epoch
2. Calculate >50 % >3 spike foci by determining if 10 or more 15 s pages in a 5 min epoch include >8/15 one-second bins with a spike

**Amplitude Rules:**

1. Peak-to-peak amplitude on a longitudinal bipolar montage, refers to background waves and excludes 1) the slow wave of a preceding spike and the field of these waves in other channels, 2) hypnagogic patterns, and 3) arousal rhythms
2. Waves must be common: present at least once in 10 or more 15 s pages in a 5 min epoch

3. Abnormal high amplitude:

a. >200 μv: Fp1-F7, F7-T3, Fp1-F3, F3-C3, C3-P3, Fp2-F4, F4-C4, C4-P4, C4-P4, Fp2-F8, F8-T4

b. >300 μv: T3-T5, T4-T6

c. Excluded: Fz-Cz, Cz-Pz, T5-O1, P3-O1, P4-O2, T6-O2

**Grouped Multifocal Spikes Definition:**

1. At least 2 different spike foci in each hemisphere within a fairly well-delineated group (may include midline)
2. For hemispheric grouping, at least 3 different spike foci in one hemisphere (may include midline) within a fairly well-delineated group

**Paroxysmal Voltage Attenuation Definition:**

1. Definite sudden change from ongoing background activities appearing as a relative attenuation most often lasting one second (but may last several seconds), often occurring after an epileptic discharge, and usually diffuse (but may be present in only one hemisphere)

**Remission Rules:**

1. Pretreatment score of 4 or 5, must improve to <3
2. Pretreatment score of 3, must improve to <2

**Supplementary Table S2.** Patient-level comparison of scalp HFO characteristics between BASED 4 and 5 within the IESS cohort

| Scalp HFO Characteristic | BASED 4 (n = 9)* | BASED 5 (n = 21)* | Hodges–Lehmann difference (5 − 4)† | Cliff's δ† | Brunner–Munzel *p* value |
|---|---|---|---|---|---|
| Frequency (Hz) | 133.3 (125.0 – 139.6) | 117.7 (107.1 – 125.0) | -15.68 | -0.624 | < 0.001 |
| Duration (msec) | 29.0 (18.5 – 31.0) | 35.0 (23.0 – 39.0) | 5.5 | 0.328 | 0.185 |
| Amplitude (Z-score) | 11.23 (9.265 – 15.64) | 10.89 (9.85 – 11.92) | -0.37 | -0.079 | 0.771 |
| Cycle count (cycles) | 4.25 (4.20 – 4.38) | 4.645 (4.50 – 4.82) | 0.32 | 0.513 | 0.041 |

Abbreviations: BASED, burden of amplitudes and epileptiform discharges; HFO, high-frequency oscillations; IESS, infantile epileptic spasms syndrome.

* Data are expressed as median (25th–75th percentiles).

† Positive Hodges–Lehmann differences and positive Cliff's δ values indicate higher values in the BASED 5 group, whereas negative values indicate lower values in the BASED 5 group.

**Supplementary Table S3.** Event-level comparison of scalp HFO characteristics between BASED 4 and 5 within the IESS cohort

| Scalp HFO Characteristic | Estimate (BASED 5 vs 4) | 95% CI | *p*[§] | FDR-adjusted *p*[‖] |
|---|---|---|---|---|
| Frequency (Hz) | -16.02[*] | -28.66 to -3.38 | 0.013 | 0.039 |
| Duration (fold-change) | 1.21[†] | 0.94 to 1.56 | 0.141 | 0.211 |
| Amplitude (Z-score) | -0.50[*] | -2.05 to 1.06 | 0.531 | 0.531 |
| Cycle count (fold-change) | Not reliably estimable[†,‡] | – | – | – |

Abbreviations: BASED, burden of amplitudes and epileptiform discharges; CI, confidence interval; FDR, false discovery rate; HFO, high-frequency oscillations.

[*] Estimates are expressed as absolute differences (Hz, Z-score).

[†] For duration and cycle count, values were log-transformed before modeling; therefore, effect estimates are presented as multiplicative ratios rather than absolute differences.

[‡] The cycle count model showed boundary/singularity-related numerical instability and was therefore retained for transparency but not interpreted inferentially.

[§] P-values are derived from linear mixed-effects models with patient-level random intercepts and age as a covariate.

[‖] FDR-adjusted *p* values were calculated across the three reliably estimable event-level scalp HFO characteristics: frequency, amplitude, and duration.
